# Theoretical-practical training of students from high school to care for cardiac arrest: a prospective study

**DOI:** 10.1186/cc14668

**Published:** 2015-09-28

**Authors:** Vanessa F Marçolla, Edna R Santos, Ivana PB Aragão, Karina R Silva, Kleison P Silva, Lorhana VB Oliveira, Marcio Canedo, Raphaela B Gomes

**Affiliations:** 1 Rio de Janeiro State Government, Santa Cruz, Rio de Janeiro, Rio de Janeiro, Brazil

## Introduction

Cardiovascular diseases are the leading cause of death in the world, and sudden cardiac arrest is a major contributor to this index. Training reduces ignorance and fear, increasing safety to recognize that the victim is not breathing properly, so as to trigger help and start CPR as soon as possible.

## Objective

To apply theoretical-practical training to a vocational public high school, so as to work correctly, quickly and safely before cardiopulmonary arrest, resuscitation maneuvers running efficiently, in order to save lives.

## Methods

This study was designed as a prospective investigation of 1800 students from a vocational public high school, between 2012 and 2013. The program of theoretical and practical training lasts 2 hours. Each student attends a lecture with a video on the subject for 30 minutes after 30 minutes of classroom practice. Then, using a practical training mannequin, they are assessed through a performance checklist. A questionnaire was distributed before the start of training to see whether the student had prior knowledge about a rescue in the event of cardiac arrest.

## Results

More than 50 % did not have any knowledge about the subject. This evaluation showed that after 2 hours of training and analyzing the performance checklists: 85 % knew how to perform the procedures and call for help effectively, 30 % were able to recognize the absence of breathing, and 35 % positioned themselves and began chest compressions as recommended form.

## Conclusion

Students from the school showed that 90 % of adolescents when trained are able to act at the scene of a cardiac arrest, multiplying the knowledge to family and community to save lives. However, according to the international recommendations, retraining as an ideal does not exceed 2 years.

